# Omega-3 Polyunsaturated Fatty Acids Protect against High-Fat Diet-Induced Morphological and Functional Impairments of Brown Fat in Transgenic *Fat-1* Mice

**DOI:** 10.3390/ijms231911903

**Published:** 2022-10-07

**Authors:** Lei Hao, Yong-Hui Nie, Chih-Yu Chen, Xiang-Yong Li, Kanakaraju Kaliannan, Jing X. Kang

**Affiliations:** 1 Laboratory for Lipid Medicine and Technology (LLMT), Department of Medicine, Massachusetts General Hospital and Harvard Medical School, Boston, MA 02129, USA; 2 Department of Nursing and Allied Health Professions, Indiana University of Pennsylvania, Indiana, PA 15705, USA

**Keywords:** obesity, omega-3 fatty acids, *fat-1* transgenic mice, brown adipose tissue, energy homeostasis, thermogenesis, lipopolysaccharide, inflammation

## Abstract

The role of omega-3 polyunsaturated fatty acids (n-3 PUFAs) in the regulation of energy homeostasis remains poorly understood. In this study, we used a transgenic *fat-1* mouse model, which can produce n-3 PUFAs endogenously, to investigate how n-3 PUFAs regulate the morphology and function of brown adipose tissue (BAT). We found that high-fat diet (HFD) induced a remarkable morphological change in BAT, characterized by “whitening” due to large lipid droplet accumulation within BAT cells, associated with obesity in wild-type (WT) mice, whereas the changes in body fat mass and BAT morphology were significantly alleviated in *fat-1* mice. The expression of thermogenic markers and lypolytic enzymes was significantly higher in *fat-1* mice than that in WT mice fed with HFD. In addition, *fat-1* mice had significantly lower levels of inflammatory markers in BAT and lipopolysaccharide (LPS) in plasma compared with WT mice. Furthermore, *fat-1* mice were resistant to LPS-induced suppression of UCP1 and PGC-1 expression and lipid deposits in BAT. Our data has demonstrated that high-fat diet-induced obesity is associated with impairments of BAT morphology (whitening) and function, which can be ameliorated by elevated tissue status of n-3 PUFAs, possibly through suppressing the effects of LPS on inflammation and thermogenesis.

## 1. Introduction

Obesity has become a worldwide health challenge due to dramatic increase in prevalence and its related complications, such as type 2 diabetes, non-alcoholic fatty liver diseases, cardiovascular diseases, and certain types of cancers [1,2]. New evidence suggests that obesity is a risk factor for coronavirus disease-19 (COVID-19) [3]. The exact causes of obesity remain controversial and are still under investigation [4]; however, it is believed that multi-factors, including genetic makeup [5], physical activity [6], diet, and some environmental and social factors [4], contribute to the development of obesity [7]. In particular, diet is one of the major players in the development of obesity [8].

In the past 30 years, the energy intake from total fat and saturated fat has continuously decreased in the Western diet, while the intake of omega-6 (n-6) polyunsaturated fatty acid (PUFAs) increased and the omega-3 (n-3) PUFAs decreased [9,10]. As a consequence, the ratio of n-6/n-3 PUFAs has increased from 1:1 during evolution to 20:1 today or even higher [10]. Interestingly, the change of n-6/n-3 PUFAs ratio parallels a significant increase in the prevalence of overweight and obesity [10]. Accumulating evidence suggests that an unbalanced n-6/n-3 PUFAs ratio contribute to the development of atherosclerosis, obesity, and diabetes [9,11]. In a prospective study, Wang et al. found that n-3 PUFAs in erythrocyte membrane phospholipids are inversely associated, while n-6/n-3 PUFA ratio is positively associated with longitudinal weight gain [12]. However, randomized controlled trials in humans examining the relationship between n-3 PUFAs supplementation and body weight have produced conflicting results due to many factors, such as differences in study design, dosage, timing, duration of n-3 PUFAs administration, n-6/n-3 PUFA ratio of the background diet, use of other supplements in addition to n-3 PUFAs, and demographics of the study population [10]. Numerous animal studies have demonstrated that n-3 PUFAs and n-6 PUFAs may have opposing effects on body fat gain through regulation of adipogenesis [13], lipid homeostasis [14], brain–gut–adipose tissue axis [15], and systemic inflammation [16]. A most recent study using transgenic *fat-1* mouse model revealed that endogenous elevation of n-3 PUFAs and reduction in n-6 PUFAs significantly improved obesity, diabetes, hypercholesterolemia, and hepatic steatosis induced by a high-fat diet, suggesting that balanced n-6/n-3 PUFAs ratio represents a novel therapeutic approach to treat obesity and its related disorders [17].

Adipose tissue can be classified into white adipose tissue (WAT) and brown adipose tissue (BAT). WAT mainly stores energy in the form of triglycerides, whereas BAT is a major organ involved in nonshivering thermogenesis in mammals [18]. A number of studies have demonstrated that activation of BAT may protect again obesity [19,20]. Recent finding using ^18^F-FDG PET/CT technology revealed the existence of activity of BAT in adult humans [21,22,23,24]. Therefore, brown fat is emerging as a new target for fighting obesity and metabolic syndrome [19,25,26]. One early study by Ohno et al. revealed that docosahexaenoic acid (DHA) was decreased in both BAT and plasma of rats in cold acclimation, suggesting that DHA in BAT is involved in thermogenesis [27]. Accordingly, several studies have shown that fish oil supplementation could upregulate thermogenic markers, suggesting that an elevated thermogenesis may contribute to the body weight-lowering effect of n-3 PUFAs [28,29,30]. In addition, one study showed that n-3 PUFAs increased brown adipose tissue mitochondrial GDP binding and cytochrome c oxidase activity without affecting UCP-1 content [31]. Therefore, it is very important to understand how the dietary factor, n-3 PUFAs, regulates brown fat activity in order to come up with new therapeutic regime for prevention and treatment of obesity.

Traditionally, fish oil (rich in n-3 PUFAs) and plant seed/vegetable oil (rich in n-6 PUFAs) were used to modify diets. However, these oils contain different levels of other components, such as saturated fatty acids, monounsaturated fatty acids, cholesterol, antioxidants, contaminants, and other bioactive compounds which affect the study outcome of interest. In this study, we investigated the effect of n-3 PUFAs on brown fat by utilizing *fat-1* transgenic mice, which carry a *fat-1* gene from the roundworm *Caenorhabditis elegans* and are able to convert n-6 PUFAs to n-3 PUFAs when the mice are fed a diet high in n-6 PUFAs [32]. The use of *fat-1* transgenic mice as a model of elevated tissue status of n-3 PUFAs can eliminate the confounding factors of diet (33). Our study demonstrated that elevated tissue levels of n-3 PUFAs can improve brown fat whitening and dysfunction induced by high-fat diet through, at least in part, lowering the level of LPS in circulation. These effects of n-3 PUFAs on brown fat may contribute to their anti-obesity effects (less body weight gain and fat accumulation observed in this study).

## 2. Results

### 2.1. Transgenic Fat-1 Mice Were Resistant against Obesity Induced by a High-Fat Diet

**Figure 1:**
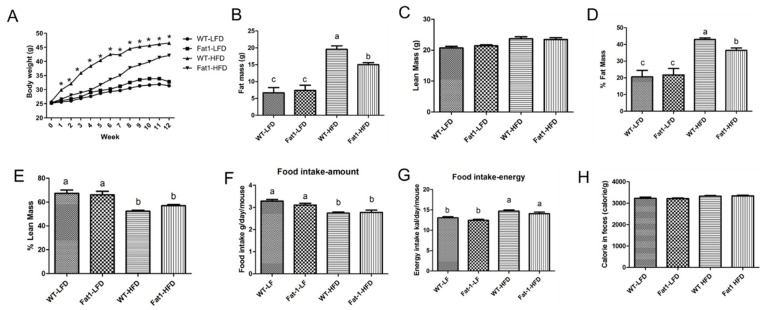
**Transgenic *fat-1* mice were resistant against obesity induced by a high-fat diet.** (**A**) body weight gain chart of *fat-1* and WT mice fed either a low-fat diet or a high-fat diet, (**B**) fat mass measured by MRI, (**C**) lean mass measured by MRI, (**D**) fat mass expressed a percentage of total body weight, (**E**) lean mass expressed as percentage of total body weight, (**F**) food intake expressed the amount of food consumption by each mouse each day, (**G**) energy intake was calculated based on energy density of corresponding to different diet and food intake, (**H**) residual energy in feces. Values represent mean or mean ± S.E.M. Error bars represent S.E.M., and different letters indicate significant difference. * represents significant difference between *fat-1* mice and WT mice fed a high-fat diet at indicated week. A significant difference was defined as *p* < 0.05.

We fed *fat-1* mice and their WT littermates with a high-fat diet (HFD) or a low-fat diet (LFD) for 12 weeks to assess the effects of endogenous omega-3 fatty acids on the development of obesity. As depicted in Figure 1A–C, there was no difference in body weight, fat mass, and lean mass between WT mice and *fat-1* mice when fed with LFD. Intake of HFD for 12 weeks induced obesity in WT mice as shown by significant weight gain, and fat mass (Figure 1A–D). By contrast, *fat-1* mice were partially resistant against HFD-induced obesity as evidenced by lower weight gain, and less fat mass (Figure 1A–E). Mice fed LFD had higher absolute amount of food intake than mice fed HFD (Figure 1F). When food intake was converted to total energy intake, mice fed HFD had higher energy intake than mice fed LFD, but there was no difference between WT and *fat-1* fed the same diet (Figure 1G). In addition, calorie residue in feces was the same among all groups (Figure 1H).

**Figure 2:**
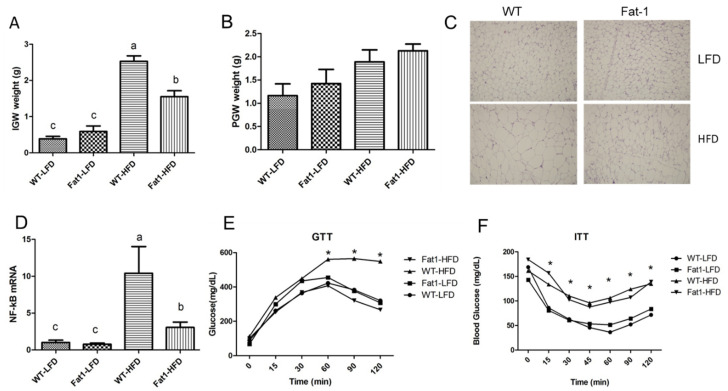
**Transgenic *fat-1* mice had improved adipose tissue inflammation and glucose tolerance.** (**A**) the weight of inguinal adipose tissue (IGW), (**B**) the weight of perigonadal adipose tissue (PGW), (**C**) H and E staining of IGW, (**D**) NF-kB gene expression in IGW, (**E**) intraperitoneal glucose tolerance test on mice injected with 0.75 g glucose per kg after overnight fast, (**F**) insulin tolerance test was evaluated throughout and injected with 0.75U insulin per kg after 6 h of fasting. Values represent mean ± S.E.M. Error bars represent S.E.M., and different letters indicate significant difference. * represents significant difference between *fat-1* mice and WT mice fed high-fat diet at indicated time points. A significant difference was defined as *p* < 0.05.

The weight of inguinal adipose tissue (IGW) was highest in WT mice among all groups (Figure 2A), whereas no significant difference was found in perigonadal adipose tissue (PGW) (Figure 2B). H and E staining of IGW also indicated that WT mice fed HFD have largest adipocyte size (Figure 2C). Along with adiposity, HFD feeding significantly induced gene expression of NF-kB, a marker of inflammation in IGW (Figure 2D). Conversely, *fat-1* mice were protected against the induction of gene expression of NF-kB in IGW (Figure 2D). Consistent with reduced adiposity, glucose tolerance test was better in *fat-1* mice fed HFD at week 8 compared with WT mice (Figure 2E). Despite the difference in glucose tolerance, both WT and *fat-1* mice had similar response to insulin challenge (Figure 2F), suggesting that *fat-1* mice may have decreased glucose production in the liver, rather than an increase in glucose uptake by extra-hepatic tissues, such as muscle and adipose tissue.

### 2.2. Fat-1 Mice Were Largely Protected from HFD-Induced Fatty Liver

**Figure 3:**
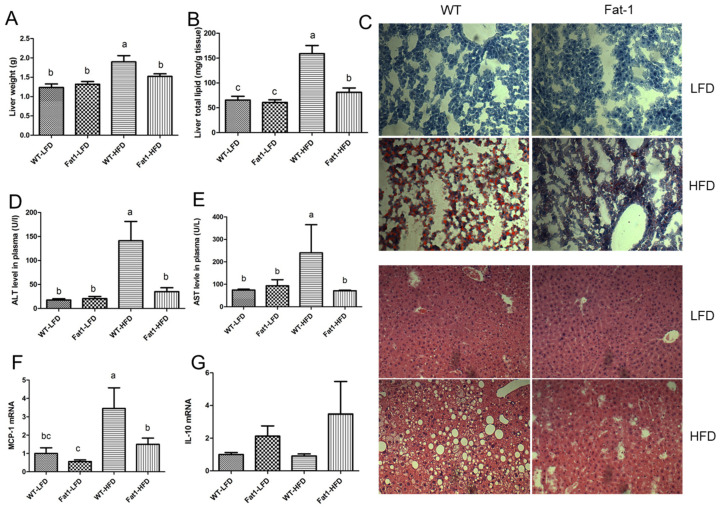
***Fat-1* mice were protected against high-fat diet-induced nonalcoholic fatty liver disease.** (**A**) the weight of liver, (**B**) total lipid extracted by using Folch method and weighted by gravity, (**C**) Oil Red O staining of frozen section of liver and H and E staining of liver samples fixed with PBS Buffer 4% formalin, (**D**) plasma ALT, (**E**) plasma AST, (**F**) liver MCP-1 gene expression, (**G**) liver IL-10 gene expression. Values represent mean ± S.E.M. Error bars represent S.E.M., and different letters indicate significant difference. A significant difference was defined as *p* < 0.05.

HFD feeding for 12 weeks led to the development of nonalcoholic fatty liver in WT mice as evidenced by increased total liver mass (Figure 3A), hepatic lipid (Figure 3B), and massive lipid droplets accumulation shown by both Oil Red O and H and E staining (Figure 3C). Conversely, *fat-1* mice were largely protected from HFD-induced fatty liver as evidenced by normal level of total liver lipid content and liver histology (Figure 3B,C). In addition, plasma ALT and AST levels were significantly higher in WT fed a high-fat diet compared with *fat-1* mice (Figure 3D,E). Furthermore, real-time PCR showed that macrophage infiltration marker, MCP1, was significantly higher in WT mice, suggesting that *fat-1* mice can suppress hepatic inflammation (Figure 3F). Consistent with the anti-inflammatory effects of n-3 PUFAs, real-time PCR showed that the anti-inflammatory cytokine, IL-10 tended to be higher in *fat-1* mice fed on either high-fat diet or low-fat diet compared to WT mice (Figure 3G).

### 2.3. Endogenous n-3-PUFAs in Fat-1 Mice Protected against HFD-Induced Morphological and Functional Impairments of Brown Fat

**Figure 4:**
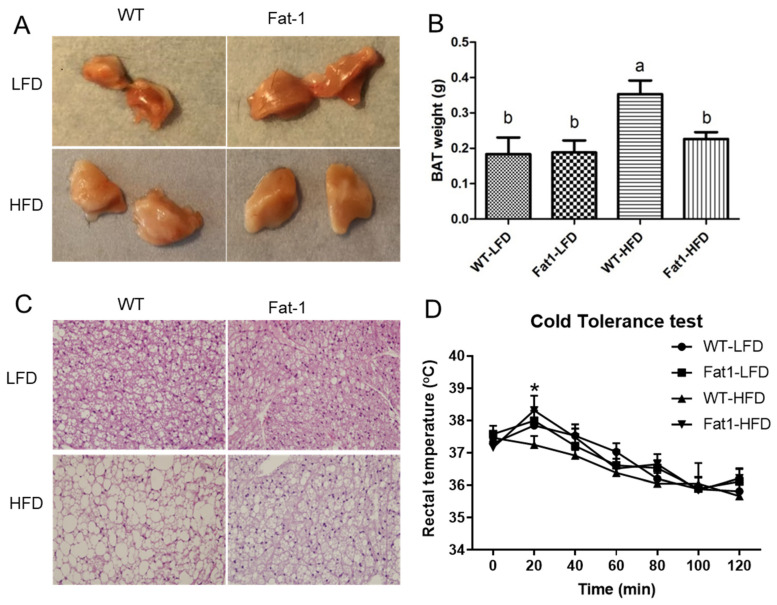
**Endogenous n-3 PUFAs in *fat-1* mice protected against HFD-induced morphological and functional impairments of brown fat.** (**A**) appearance of brown fat after dissection, (**B**) the weight of brown fat, (**C**) representative H and E staining of brown fat tissues, (**D**) after food deprivation, rectal temperatures during cold exposure were recorded in 20 min intervals in a cold room (4 °C). Values represent mean ± S.E.M. Error bars represent S.E.M., and different letters indicate significant difference. * represents significant difference between *fat-1* mice and WT mice fed a high-fat diet at indicated time points. A significant difference was defined as *p* < 0.05.

HFD feeding led to a profound “whitening” change in BAT appearance only in WT mice but not in *fat-1* mice (Figure 4A, B). This appearance change was due to the enlarged lipid droplets in brown fat cells (morphologically, they looked like white fat cells) (Figure 4C). In contrast, the morphology of brown fat cells in *fat-1* mice looked quite normal (Figure 4C). We also performed a cold tolerance test, which reflects the activity of thermogenesis of BAT. We found that *fat-1* mice fed HFD could better tolerate to the cold exposure at 20 min than WT mice fed HFD (Figure 4D).

**Figure 5:**
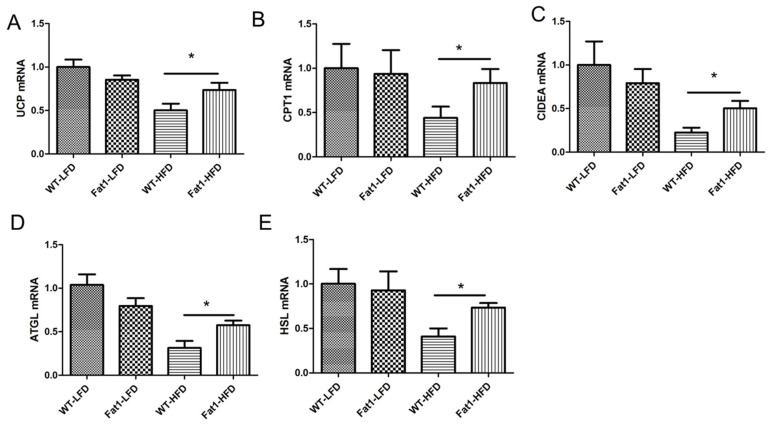
***Fat-1* mice were resistant to high-fat diet-induced downregulation of genes related to thermogenesis and lipolysis in BAT.** (**A**)—gene expression of UCP-1, (**B**)—gene expression of CPT-1, (**C**)—gene expression of CIDEA, (**D**)—gene expression of ATGL, (**E**)—gene expression of HSL. Error bars represent S.E.M., and * represent significant difference between *fat-1* mice and WT mice fed a high-fat diet. A significant difference was defined as *p* < 0.05.

Next, we investigated the expression of the genes involved in brown fat functional activity by real-time PCR. We found that UCP-1, CPT1, CIDEA, ATGL, and HSL genes were significantly downregulated in the BAT of WT mice fed HFD (Figure 5A–E), whereas the levels of these genes were significantly higher in the BAT of *fat-1* mice.

**Figure 6:**
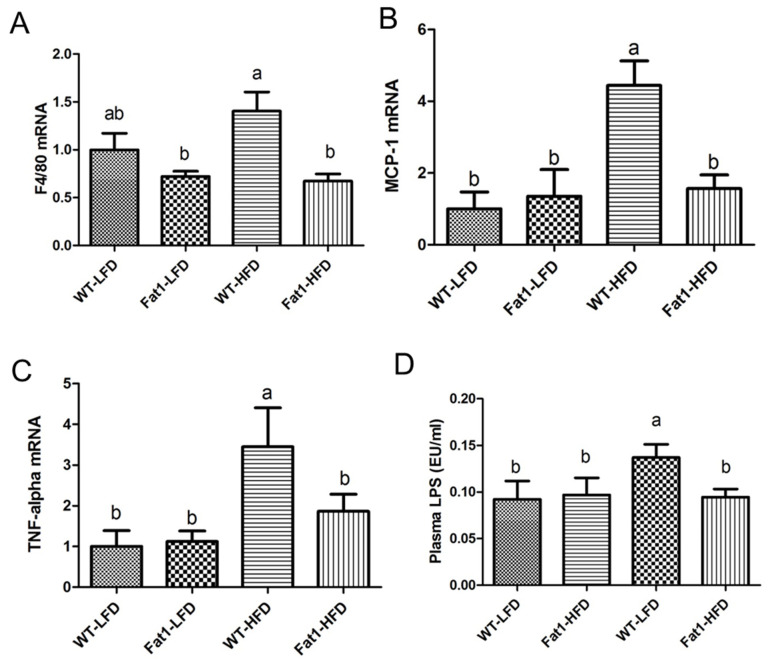
***Fat-1* mice had lower levels of inflammation markers in brown fat.** (**A**) gene expression of F4/80, (**B**) gene expression of MCP-1, (**C**) gene expression of TNF-alpha, (**D**) plasma LPS. Error bars represent S.E.M., and different letters indicate significant difference. A significant difference was defined as *p* < 0.05.

In addition, we measured inflammation markers in brown fat, such as F4/80, MCP-1, and TNF-α. All these inflammatory cytokines were higher in brown fat of WT mice compared with *fat-1* mice (Figure 6A–C). We then measured plasma LPS and found that LPS level in WT fed HFD was the highest among all groups, while the LPS level in *fat-1* mice fed HFD was close to that in mice fed a low-fat diet (Figure 6D).

### 2.4. Fat-1 Mice Were Resistant to LPS-Induced Suppression of UCP1 and PGC-1 Expression and Lipid Deposits in BAT

**Figure 7:**
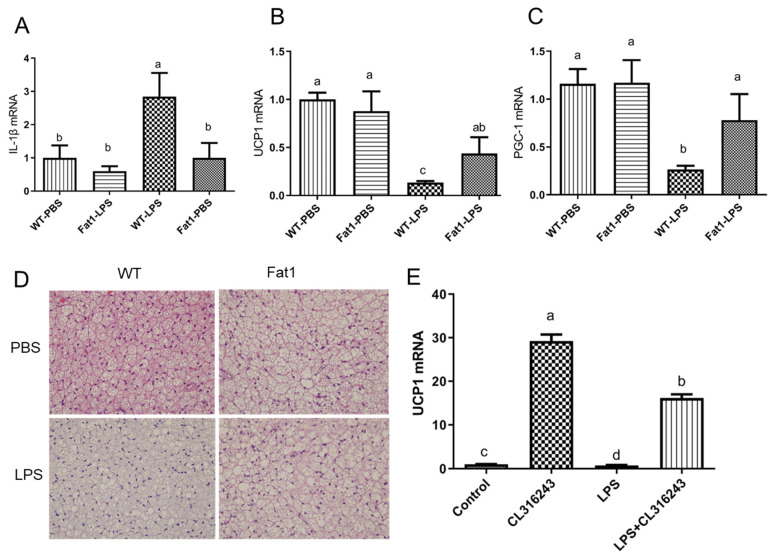
**Effect of LPS on inflammation and thermogenic gene expression in BAT of *fat-1* mice and in cultured brown fat cells in vitro.** (**A**) gene expression of IL-1beta, (**B**) gene expression of UCP-1, (**C**) gene expression of PGC-1, (**D**) H and E staining of brown fat tissue in WT and *fat-1* mice treated with PBS or LPS (**E**) UCP-1 gene expression in immortal brown fat cells treated with indicated conditions. Error bars represent S.E.M., and different letters indicate significant difference. A significant difference was defined as *p* < 0.05.

To examine if LPS can suppress brown fat activity and if *fat-1* mice can be protected against the LPS-induced suppression of brown fat activity, both WT and *fat-1* mice received intraperitoneal injection of LPS every other day for 2 weeks. As expected, LPS stimulated expression of inflammation markers, such as IL-1beta, in brown fat of WT mice. However, *fat-1* mice were protected from LPS-induced inflammation in BAT (Figure 7A). We observed that LPS significantly suppressed brown fat activity markers, such as UCP-1 and PGC-1, in WT mice but not in *fat-1* mice (Figure 7B,C). H and E staining of BAT showed that brown fat cells in WT mice treated with LPS have larger lipid droplets than that in *fat-1* mice treated with LPS (Figure 7D).

### 2.5. LPS Suppressed the Upregulation of UCP1 Induced by the β3-Adrenergic Agonist, CL-316243, in Mouse Brown Adipocytes In Vitro

To examine if long-term LPS exposure, which is the case in obesity, has a direct suppressive effect on brown fat cell activity, cultured mouse brown adipocytes were pre-incubated with LPS for 18 h before they were exposed to beta-3 adrenergic agonist, CL-316243 for 6 h. Real-time PCR data showed that UCP-1 gene expression was dramatically induced by CL-316243 alone (Figure 7E). However, pre-incubation with LPS significantly inhibited UCP-1 induction by CL-316243 (Figure 7E), suggesting a role played by LPS in modulating the function of BAT.

## 3. Discussion

In the present study, we used a *fat-1* transgenic mouse model that carries the roundworm *Caenorhabditis elegans fat-1* gene, which can covert n-6 PUFAs to n-3 PUFAs [32]. The use of the *fat-1* transgenic mouse model can eliminate the potential confounding factors derived from dietary n-3 PUFAs supplementation in most nutrition studies [33]. Although several studies have used *fat-1* transgenic mouse model to investigate the effects of n-3 PUFAs on obesity, diabetes, and NAFLD [17,34,35,36,37,38,39,40], the present study is the first using the model to specifically investigate how n-3 PUFAs regulate brown fat function. A novel finding in our study is that HFD feeding led to “whitening” in brown fat tissue in WT mice, whereas *fat-1* mice were protected against such morphology change caused by HFD feeding. 

In the current literature, most studies have investigated “browning” of white adipose tissue as an approach to combat obesity, whereas only a few studies have investigated the impact and molecular mechanisms that contribute to obesity-linked BAT dysfunction—a process that is associated with the “whitening” of this tissue [41]. Our study is the first to find that *fat-1* mice were able to resist HFD-induced “whitening” of brown fat. The mass of BAT in WT fed HFD is enlarged due to the “whitening” change in cellular content, characterized by a large single lipid droplet in brown fat cells instead of the small multilocular lipid droplets in normal BAT cells, indicating that brown fat structure and function were impaired by HFD feeding in WT mice. We further performed a cold tolerance test to examine the function of BAT in both WT and *fat-1* mice. During 120 min exposure to cold, body temperature of WT mice dropped markedly, displaying impaired adaptive thermogenesis compared with *fat-1* mice. In addition, real-time PCR data demonstrated that thermogenic markers, such as UCP-1, CPT-1, and CIDEA, were suppressed in WT mice. Furthermore, several genes related to lipolysis of BAT were significant suppressed in WT mice compared with *fat-1* mice. All these data support the notion that n-3 PUFAs can protect BAT from HFD-induced impairments of morphology and function.

A key question is that why the high-fat diet led to “whitening” change and functional impairments of brown fat. Shimizu et al. revealed that BAT “whitening” phenotype was associated with decreased expression of vascular endothelial growth factor A (VEGFA), and that targeted deletion of VEGFA in adipose tissue of nonobese mice resulted in BAT whitening [42]. We examined VEGFA expression in BAT in our study but found no difference in VEGFA expression among all groups. Two recent studies showed that inflammation is responsible for compromised adaptive thermogenesis, thereby contributing to the development of obesity [43,44]. We hypothesized that LPS, a marker of metabolic endotoxemia known to be reduced by n-3 PUFAs [45], plays a role in the HFD-induced impairments of BAT. Consistent with our hypothesis, we found that the level of LPS in plasma of WT mice was significantly higher than that of *fat-1* mice fed HFD. In addition, inflammation markers, such as F4/80, MCP-1, and TNFα, were all increased in WT mice fed HFD. Our previous study has demonstrated that elevated tissue n-3 PUFAs could modulate the gut bacteria composition, resulting in decreased LPS production and gut permeability [45]. In the present study, when we treated both WT and *fat-1* mice with LPS for 2 weeks, LPS significantly suppressed BAT’s UCP-1 expression in WT mice but not in *fat-1* mice, suggesting that endogenous n-3 PUFAs can confer a resistance to the LPS-induced suppression of adaptive thermogenesis. Consistently, our data from the in vitro study showing that the induction of UCP-1 expression by the β3-agonist, CL-316243, in immortalized brown adipocytes was markedly suppressed by pre-incubation of the cells with LPS, support the notion that LPS is a suppressor of adaptive thermogenesis.

## 4. Conclusions

**Figure 8:**
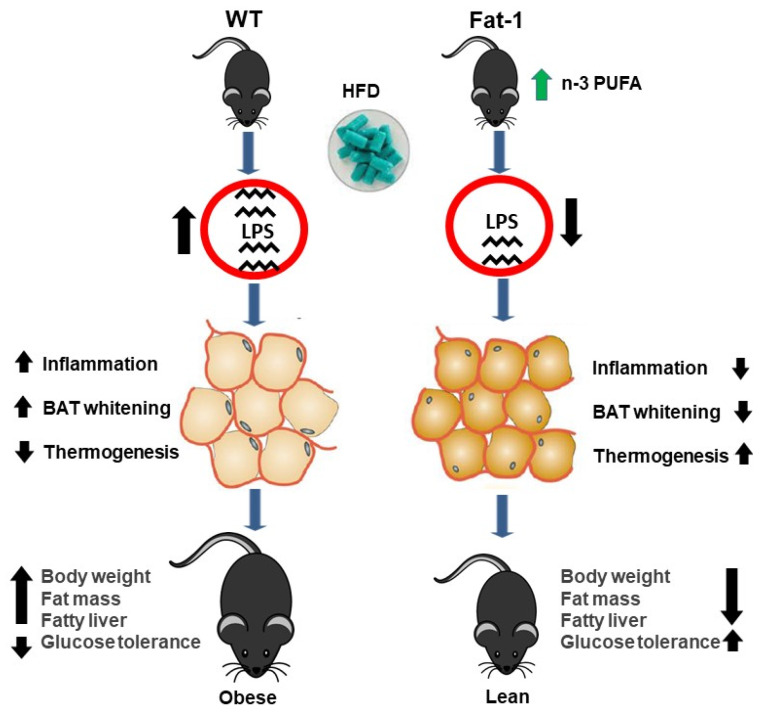
**A proposed mechanism for the anti-obesity effects of n-3 PUFAs in *fat-1* mice.** Diagram illustrates HFD may induce obesity through the impairments of BAT morphology (whitening) and function (thermogenetic activity). Elevated tissue status of omega-3 fatty acids can reduce HFD-induced increase of LPS, suppress the generation of inflammatory cytokines and thereby protect against the damages of BAT’s morphology and function, leading to the anti-obesity effects (decreased body weight, less fat mass, improved fatty liver and glucose tolerance, etc.).

This study has demonstrated that high-fat diet-induced obesity is associated with impairments of BAT morphology (whitening) and function, which can be ameliorated by elevated tissue status of omega-3 PUFAs. Our study also reveals a potential mechanism by which omega-3 PUFAs alleviate the dysfunction of BAT through suppressing the effects of LPS on inflammation and thermogenesis (Figure 8).

## 5. Materials and Methods

### 5.1. Animal Experiments

Transgenic male *fat-1* mice were generated as described elsewhere [32,33]. Briefly, the *C. elegans* omega-3 fatty acid desaturase *fat-1* gene was coupled to a β-actin promoter, which allows a broad expression of all tissues in mice. The phenotype of *fat-1* mice was confirmed according to fatty acids profile analyzed by gas chromatography (GC). The ratio of n-6/n3 in tissues of *fat-1* transgenic mouse model is close to 1:1. Transgenic *fat-1* mice and their wild type littermates were fed either a low-fat diet (1812692, TestDiet) or high-fat diet (D12492, Research Diet) for 12 weeks. Diet information can be found in Supplementary Table S1. Mice were individually housed in a humidity-controlled room under a 12 h light/dark cycle and had free access to water and food during the study. Body weight and food intake were measured weekly. Body composition, including fat mass, lean mass, and water, was assessed on the day of sacrifice by Bruker’s minispec LF 110 Body Composition Analyzer (Bruker Billerica, MA, USA). In another separate animal study, both *fat-1* and wild type mice were fed the low-fat diet and received either PBS or LPS treatment for 7 days. At termination, mice were fasted for 6 h and euthanized with carbon dioxide. Plasma and tissues were then collected and stored in −80 °C before analysis. For histological analysis, a portion of liver, inguinal WAT, and BAT were fixed with 10% phosphate-buffered formalin or embedded in Tissue-Tek^®^ O.C.T compound (Sakura Finetek, Torrance, CA, USA). All animal protocols were approved by the Institutional Animal Care & Use Committee (IACUC) of Massachusetts General Hospital (Animal protocol No: 2010N000038).

### 5.2. RNA Extraction and Real Time PCR

RNA extraction and real-time PCR were described elsewhere [34]. Briefly, total RNA was extracted from mouse liver, inguinal WAT, and BAT by using TRIzol reagents (Life Technology, Carlsbad, CA, USA). The reverse transcription reaction was performed by using an input of 1µg total RNA and iScriptTM Reverse Transcription Supermix (Catalog# 170-8840, BIO-RAD). The resultant cDNA was amplified by using iTaq Universal SYBR Green Supermix (Catalog# 172-5124, BIO-RAD) in the Agilent Mx3000P qPCR System. The PCR reaction conditions for each cycle were as follows: 95 °C for 30 s, followed by 40 cycles of at 95 °C for 5 s, 60 °C for 30 s, and 1 cycle of at 95 °C for 60 s, 55 °C for 30 s, and 95 °C for 30 s. Relative gene expression was analyzed by the 2^-ΔΔCT^ method and normalized with β-actin. Primer sequences are listed in Supplementary Table S2.

### 5.3. Histology

For Oil Red O staining, froze liver tissues embedded in Tissue-Tek^®^ O.C.T compound (Sakura Finetek, Torrance, CA, USA) were cut into 10 µm-thick sections and stained with Oil Red O as described elsewhere [35]. For H and E staining, tissues were fixed with 10% PBS buffered formalin-fixed for 48 h. Hematoxylin and eosin stained was performed by Massachusetts General Hospital (MGH Core, Boston, MA, USA).

### 5.4. Liver Total Lipid Quantification

Total lipid was extracted according to Folch method [36] and measured by gravity.

### 5.5. Cold Tolerance Test

For the cold tolerance test, the animals were subjected to a cold room (4 °C) without access to food. The rectal temperature was measured by using the BAT-12 thermometer (Physitemp Instruments, LLC, Clifton, NJ, USA). No sedation or anesthesia was used when we measured rectal temperature.

### 5.6. Intraperitoneal Insulin Tolerance Test (IPITT)

Fasting blood glucose was measured (4 h fast, blood taken from the tail vein) using a blood glucose meter (Bayer HealthCare LLC, Mishawaka, IN, USA) and Contour blood glucose test strips. Then, insulin was injected intraperitoneally (0.75 U/kg) and blood glucose was measured again at time points of 15, 30, 60 and 120 min, post injection.

### 5.7. Intraperitoneal Glucose Tolerance Test (IGTT)

Fasting blood glucose was measured (6 h fast, blood taken from the tail vein) according to manufacturers’ recommendations using a blood glucose meter (Bayer HealthCare LLC, Mishawaka, IN, USA) and Contour blood glucose test strips. Glucose was then administrated by intraperitoneal injection (25% glucose solution, 1 g/kg mice) and blood glucose was measured again at time points of 15, 30, 60 and 120 min, post injection.

### 5.8. Cell Culture

The mouse SV40T immortalized mouse brown adipocytes are gifts from Dr. Yu-Hua Tseng, Joslin Diabetes Center of Harvard Medical School. Brown adipocytes were grown in DMEM media containing 20% FBS and 1% penicillin/streptomycin in a humidified environment at 37 °C with 5% CO_2_. After reaching 100% confluence, BAT cells were subjected to induction for 2 days by adding induction media supplemented with 0.5 mM isobutylmethylxanthine, 0.5 mM dexamethasone, and 0.125 mM indomethacin. Subsequently, the cells were maintained in differentiation medium for 5–6 days until exhibiting a fully differentiated phenotype with massive accumulation of multilocular fat droplets. Experiments were carried out after incubating the cells in complete growth medium for 18 h.

### 5.9. Measurement of LPS Concentration

Plasma LPS concentrations were measured with a ToxinSensorTM Chromogenic LAL Endotoxin Assay Kit (GenScript, Piscataway, NJ, USA), following the manufacturer’s instructions. Briefly, samples were diluted 20-fold with endotoxin-free water included in the kit and incubated for 10 min at 37 °C to minimize inhibition or enhancement by contaminating proteins. LAL reagents were added to plasma and incubated at 37 °C for 60 min, and the absorbance was read at 545 nm. LPS concentration was calculated based on the standard curve.

### 5.10. Total Fecal Energy Content Analysis

To estimate nutrient absorption, we consecutively collected mouse feces for 3 days. The total energy residue in the samples of homogenized stools was measured by the Central Analytical Laboratory of the Department of Poultry Science of the University of Arkansas.

### 5.11. Statistical Analysis

Data are expressed as mean ± SEM for the number of replicates indicated. Statistical analysis was performed using two-way ANOVA or Student’s *t*-test, as appropriate (Prism 9, GraphPad Software, San Diego, CA, USA). A significant difference was defined as *p* < 0.05.

## Data Availability

Not applicable.

## Supplementary Materials

The following supporting information can be downloaded at: https://www.mdpi.com/article/10.3390/ijms231911903/s1
