# Supplementary material for: Omega-3 Polyunsaturated Fatty Acids Protect against High-Fat Diet-Induced Morphological and Functional Impairments of Brown Fat in Transgenic Fat-1 Mice
Source: Int J Mol Sci. 2022 Oct 7;23(19):11903. doi: 10.3390/ijms231911903 (PMC9570395; doi:10.3390/ijms231911903)
Supplement: Supplementary file 1 [file ijms-23-11903-s001.zip › ijms-1932286-supplementary.pdf]

International Journal of Molecular Sciences

MDPI

*Supplementary Tables*

**Table S1.** Ingredients of diets.

| | LFD[1] | HFD[1] |
|---|---|---|
| **Ingredient (g/kg)** | | |
| Casein | 235.3 | 258.4 |
| L-Cystine | 0.9 | 3.9 |
| Corn starch | 0.0 | 0.0 |
| Maltodextrin | 103.2 | 161.5 |
| Sucrose | 443.8 | 88.9 |
| Cellulose | 58.8 | 64.6 |
| Corn oil | 100 | 0.0 |
| Soybean oil | 0.0 | 32.3 |
| Lard | 0.0 | 316.6 |
| Mineral Mix | (AIN-76) 41.2 | (S10026) 12.9 |
| Vitamin Mix | (AIN-76A)11.8 | (V10001) 12.9 |
| Choline bitartrate | 2.4 | 2.6 |

[1] Abbreviations are: LFD, low fat diet; HFD, high fat diet.

**Table S2.** Primer sequences used in this study.

| Gene Symbol | Gene Name | F, Forward primer<br>R, Reverse primer |
|---|---|---|
| B-actin | | F: 5'-GACGGCCAGGTCATCACTAT -3'<br>R: 5'-ACATCTGCTGGAAGGTGGAC -3' |
| *UCP-1* | | F: 5'- AGGCTTCCAGTACCATTAGGT -3'<br>R: 5'- CTGAGTGAGGCAAAGCTGATTT -3' |
| *TNFα* | | F: 5'- GACCCCTTTACTCTGACCCC -3'<br>R: 5'- AGGCTCCAGTGAATTCGGAA -3' |
| *PGC1α* | | F: 5'- TATGGAGTGACATAGAGTGTGCT-3'<br>R: 5'- CCACTTCAATCCACCCAGAAAG-3' |
| *CIDEA* | | F: 5'- TGCTCTTCTGTATCGCCCAGT -3'<br>R: 5'- GCCGTGTTAAGGAATCTGCTG-3' |
| *MCP-1* | | F: 5'- AGAAGGAATGGGTCCAGACA -3'<br>R: 5'- AAAATGGATCCACACCTTGC -3' |

| | |
|---|---|
| *F4/80* | F: 5'- CCATTGCCCAGATTTTCATC -3'<br>R: 5'- TCCTGGAGTTTGGTTCCATC -3' |
| *ADRB3* | F: 5'- AACTCTCCAACGCTCCAGAA -3'<br>R: 5'- GATGGTCCAAGATGGTGCTT -3' |
| *VEGFA* | F: 5'- TCTCTTGGGTGCACTGGACC-3'<br>R: 5'- GTTACAGCAGCCTGCACAGC -3' |
| *HSL* | F: 5'- TCATGGCTCAACTCCTTCCT -3'<br>R: 5'- ATTTTTGGCCTCAGCCTCTT-3' |
| *ATGL* | F: 5'- AGGCAGGAGGGAAAGAGAGA-3'<br>R: 5'- ACCAGGTACTGGCAGATGCT -3' |
| *NF-kB* | F: 5'- CGAGGCAGCACATAGATGAA -3'<br>R: 5'- TTGCTAGACACCGTCTGTGC-3' |
| *IL-6* | F: 5'- CCGGAGAGGAGACTTCACAG-3'<br>R: 5'- GGAAATTGGGGTAGGAAGGA -3' |
| *IL-10* | F: 5'- TCCTTGGAAAACCTCGTTTG -3'<br>R: 5'- AACTGGCCACAGTTTTCAGG-3' |
| *IL-1β* | F: 5'- CAGGCAGGCAGTATCACTCA -3'<br>R: 5'- AGGCCACAGGTATTTTGTCG -3' |
